# Association of residential air pollution and green space with all-cause and cause-specific mortality in individuals with diabetes: an 11-year prospective cohort study

**DOI:** 10.1016/j.ebiom.2024.105376

**Published:** 2024-09-30

**Authors:** Chunfeng Wu, Jiangdong Liu, Yanyun Li, Luxin Qin, Ruilong Gu, Jiachen Feng, Lulu Xu, Xia Meng, Jiaxin Chen, Renjie Chen, Yan Shi, Haidong Kan

**Affiliations:** a School of Public Health, Key Lab of Public Health Safety of the Ministry of Education and NHC Key Lab of Health Technology Assessment, Fudan University, Shanghai 200032, China; b Division of Integrated Management, Shanghai Municipal Center for Disease Control and Prevention, Shanghai, 200336, China; c Division of Chronic Non-Communicable Disease and Injury, Shanghai Municipal Center for Disease Control and Prevention, Shanghai 200336, China; d Children’s Hospital of Fudan University, National Children’s Medical Center, Shanghai 201102, China

**Keywords:** Diabetes, Air pollution, Green space, Cause-specific mortality, Prospective cohort

## Abstract

**Background:**

To assess the long-term impact of residential air pollution and green space exposure on cause-specific mortality in individuals with type 2 diabetes mellitus (T2DM).

**Methods:**

This study includes 174,063 participants newly diagnosed with T2DM from a prospective cohort in Shanghai, China, enrolled between 2011 and 2013. Residential annual levels of air pollutants, including fine (PM_2.5_) and coarse (PM_2.5-10_) particulate matter, nitrogen dioxide (NO_2_), along with the normalized difference vegetation index (NDVI), were derived from satellite-based exposure models.

**Findings:**

During a median follow-up of 7.9 years (equivalent to 1,333,343 person-years), this study recorded 22,205 deaths. Higher exposure to PM_2.5_ was significantly associated with increased risks for all mortality outcomes, whilst PM_2.5-10_ showed no significant impacts. The strongest associations of PM_2.5_ were observed for diabetes with peripheral vascular diseases [hazard ratio (HR): 2.70; per 10 μg/m^3^ increase] and gastrointestinal cancer (2.44). Effects of NO_2_ became significant at concentrations exceeding approximately 45 μg/m³, with the highest associations for lung cancer (1.20) and gastrointestinal cancer (1.19). Conversely, each interquartile range increase in NDVI (0.10) was linked to reduced mortality risks across different causes, with HRs ranging from 0.76 to 1.00. The association between greenness and mortality was partly and significantly mediated by reduced PM_2.5_ (23.80%) and NO_2_ (26.60%). There was a significant and negative interaction between NO_2_ and greenness, but no interaction was found between PM_2.5_ and greenness.

**Interpretation:**

Our findings highlight the vulnerability of individuals with T2DM to the adverse health effects of air pollution and emphasise the potential protective effects of greenness infrastructure.

**Funding:**

The 6th Three-year Action Program of Shanghai Municipality for Strengthening the Construction of Public Health System (GWVI-11.1-22), the 10.13039/501100012166National Key Research and Development Program (2022YFC3702701), and the 10.13039/501100001809National Natural Science Foundation of China (82030103, 82373532).

**Research in context**

**Evidence before this study**

Type 2 diabetes mellitus (T2DM) is a major global health concern, contributing to approximately 1.6 million deaths and 75 million disability-adjusted life years in 2021. We conducted a literature search using PubMed and Google Scholar for cohort studies published before August 1, 2024. The search terms included keywords related to air pollution (“air pollution”, “air pollutant”, “fine particulate matter”, “fine particles”, “PM_2.5_”, “nitrogen dioxide”, “oxynitride”, “NO_2_”, “coarse particles”, and “PM_2.5-10_”), green space (“green space”, “greenness”, “greenery”, “vegetation natural”, “green area”, “NDVI”, “normalized difference vegetation”), mortality (“cause-specific mortality”, “cardiovascular disease”, “respiratory disease”, “cancer”, and “metabolic disease”), study types (“long-term”, “longitudinal study”, and “cohort study”) and population terms (“diabetes”, “adults with type 2 diabetes”, and “individuals with type 2 diabetes”). Our search revealed that most relevant studies have focused on general populations without T2DM at baseline. The long-term impact of residential air pollution and green space on cause-specific mortality in individuals with T2DM remains largely unknown.

**Added value of this study**

In this 11-year prospective cohort study, we enrolled 174,063 individuals newly diagnosed with T2DM between 2011 and 2013, accumulating 1,333,343 person-years of observation. Our results revealed that long-term exposure to PM_2.5_ was associated with increased cause-specific mortality risks in this vulnerable group, with PM_2.5-10_ exhibiting insignificant effects. Higher exposure to NO_2_ was also associated with increased mortality risks, though the effects were weaker than those of PM_2.5_ and became significant only beyond approximately 45 μg/m³. The effects of air pollution exceeded those reported in other large cohorts and meta-analyses within general populations. Diabetes peripheral vascular diseases and gastrointestinal cancer are the top causes of PM_2.5_-related mortality in this population, while lung cancer and gastrointestinal cancer are the top causes of NO_2_-related mortality. Increased green space exposure was linked to reduced mortality risks, partially mediated by reductions in PM_2.5_ and NO_2_ levels. There was a significant and negative interaction between NO_2_ and green space.

**Implications of all the available evidence**

Individuals with T2DM could significantly benefit from reductions in PM_2.5_ and NO_2_, as well as increased green infrastructure (especially in areas with low green space exposure), supporting targeted public health strategies and clinical interventions.

## Introduction

Diabetes is a chronic health condition that poses a significant challenge to healthcare systems worldwide.^1^ In 2021, the global population with diabetes reached around 529 million, with an age-standardized prevalence of approximately 6.1%.^2^ Notably, with an estimated 12.4% prevalence of diabetes in 2018,^3^ China ranks among the countries with the highest number of individuals with diabetes globally and allocates the second-highest amount of resources to manage diabetes and its complications.^4^ Given the significant prevalence of diabetes and the heightened risk of mortality from various diseases faced by individuals with diabetes, it becomes imperative to investigate potential risk and protective factors within populations with diabetes rather than focusing solely on the general population, which could facilitate the evidence-based development of targeted strategies to enhance the prognosis and overall quality of life for individuals with diabetes.

According to the Global Burden of Diseases Study, air pollution was identified as the third leading contributor (20%) to diabetes-related deaths in 2019.^5^ Epidemiological studies have provided ample evidence linking air pollution exposure to increased cause-specific mortality within the general population,^6^, ^7^, ^8^, ^9^, ^10^, ^11^, ^12^, ^13^, ^14^ including mortality from diabetes and diabetic complications.^6^^,^^7^ Accordingly, we can reasonably assume that individuals with diabetes may face heightened mortality risks from various diseases due to ambient air pollution. Quantifying the effects of various air pollutants on cause-specific mortality in this population becomes paramount in verifying this hypothesis. To date, only one cohort study has explored cumulative transition probabilities from type 2 diabetes mellitus (T2DM) to all-cause mortality.^15^ However, no cohort study has quantified the effects of air pollutants on cause-specific mortality within populations with T2DM.

Several epidemiological studies have suggested that exposure to green spaces is associated with lower mortality from non-communicable diseases,^16^, ^17^, ^18^, ^19^, ^20^, ^21^, ^22^, ^23^, ^24^ including T2DM.^17^ This prompts further investigation into quantifying the mitigating effects of increased access to green spaces on the mortality risk from various major diseases among individuals with T2DM, thereby contributing to the implementation of precision medicine. However, there is limited research investigating the potentially beneficial impact of green space exposure on various health outcomes within populations with T2DM. Additionally, greenness might reduce air pollution levels by filtering out particulate matter and nitrogen oxides.^25^^,^^26^ Yet, the understanding of the mediating effect of air pollution on relationships between greenness and mortality in populations with T2DM remains largely unknown.

Therefore, we conducted a prospective cohort study among individuals newly diagnosed with T2DM to quantify their vulnerability to the effects of long-term exposure to air pollution and green spaces. We further explored potential pathways through which green spaces may exert beneficial effects on reducing mortality risks through mediation analysis, and also explored potential interactions of green space and air pollutants.

## Methods

### Study design and population

This study was based on a prospective cohort, established by the Shanghai Standardized Diabetes Management System, which registered nearly all diagnosed cases of T2DM in Shanghai, including newly diagnosed cases identified through community-based screenings and routine outpatient visits.^27^ The Shanghai Municipal Center for Disease Control and Prevention (SCDC) implemented a quality control process involving the random selection of a proportion of patients across 16 district-level CDCs for conducting annual telephone and face-to-face investigations. A more specific description is available in Supplementary Method S1 and our previous studies.^28^^,^^29^ T2DM was diagnosed based on (1) fasting plasma glucose levels of ≥7.0 mmol/L and/or (2) a 2-h glucose level (after a 75 g oral glucose tolerance test) of ≥11.1 mmol/L, or (3) the use of glucose-lowering medication according to the 1998 World Health Organization criteria.^30^ Meeting at least one of these three criteria suggests a diagnosis of diabetes. All diabetes diagnoses and classifications, including T2DM, were made by clinical endocrinologists, and individuals with type 1 diabetes were not included. From 2011 to 2013, 183,158 individuals newly diagnosed with T2DM, aged over 20 years, were enrolled. Outcomes were defined as any cause of death until December 31, 2021.

For this analysis, we excluded participants without any follow-up records after enrollment (N = 3103), and those with missing exposure or baseline covariate data (N = 1697 and 3,316, respectively). Participants with coding errors in covariate information were also excluded (N = 979), including cases where the date of diabetes diagnosis was later than the enrollment date, individuals under 20 years old, and cases where the duration of diabetes exceeded the individual’s age. Consequently, 174,063 participants were included in the final analysis (Supplementary Fig. S1). The missing exposure data were primarily due to incomplete address information. Additionally, we compared the exposure levels of participants with missing covariate data to those included in the study, and found no significant differences (Supplementary Table S1).

### Mortality data

We obtained cause-specific death data that was extracted from the Shanghai death registry. Causes of death were assessed based on the primary diagnosis coded by ICD-10 (International Classification of Diseases, 10th revision), encompassing all causes, cardiovascular diseases (CVD; I00–I99), ischemic heart disease (IHD; I20–I25), stroke (I60–I69), respiratory diseases (J00-J98), chronic obstructive pulmonary disease (COPD; J41-J44), cancer (C00–C97), gastrointestinal cancer (C15–C26), lung cancer (C34), metabolic diseases (E00-E90), T2DM (E11), and diabetes with peripheral vascular diseases (PVD; E10.5, E11.5, E12.5, E13.5, and E14.5). These causes were selected based on established epidemiological associations with air pollutants within the general population,^31^, ^32^, ^33^, ^34^ as well as their substantial contribution to mortality rates among individuals with diabetes.^35^

### Residential exposure to air pollutants and greenness

Residential exposure to air pollutants, including ambient fine particulate matter (PM_2.5_), inhaled particulate matter (PM_10_), and nitrogen dioxide (NO_2_), was evaluated by validated satellite prediction models based on random forest algorithm at a resolution of 1 × 1 km.^36^, ^37^, ^38^ The cross-validated R^2^ values of the models were 0.86, 0.87, and 0.75 for PM_2.5_, PM_10_, and NO_2_, respectively. Coarse particulate matter (PM_2.5-10_) levels were calculated as the differences between PM_2.5_ and PM_10_.

Residential surrounding greenness was characterized using the Normalized Difference Vegetation Index (NDVI), derived from Moderate Resolution Imaging Spectroradiometer (MODIS) remote sensing images.^39^ NDVI values range from −1 to 1, with higher values indicating greater greenness. NDVI data were preprocessed by leveraging quality accuracy data to exclude pixels affected by cloud cover or snow, with values bounded to be greater than 0 to account for the influence of water bodies.^40^ NDVI data were obtained at a spatial resolution of 250 m and a temporal resolution of 16 days.

By matching the exposures within the nearest grid cells of each participant’s residential address, we computed the prior 1-year average exposures for each participant annually over the entire follow-up period.

### Covariate data

We considered a wide range of covariates in terms of sociodemographic and behavioral characteristics in relation to diabetes, air pollution, or green space exposure, which were collected through questionnaires at baseline. These covariates included age (continuous); self-reported sex (female or male); body mass index (BMI: normal, underweight, overweight, or obese); family history of diabetes (yes, no, or unknown); drinking status [every day, sometimes (less than 1 day/month), usual (1 day/month to 6 days/week), never, or unknown]; smoking status (every day, usual but not every day, former smoker, never, or unknown); regular physical activity (yes or no); employed status (employed or unemployed); and medication compliance (regular, irregular, or none). Socioeconomic indicators, including gross domestic product (GDP) data at the 1 km grid level (continuous) and district-level number of doctors per 10,000 people, were also matched to each participant’s residential address. The selected covariates were verified using a directed acyclic graph (Supplementary Fig. S2). Baseline comorbidities were also considered, including diabetic CVD (1.14%), diabetic neuropathy (0.15%), and metabolic diseases (0.02%; including diabetic ketoacidosis, hyperosmolar hyperglycemic state, and diabetic lactic acidosis).

### Statistical analysis

We fitted time-dependent Cox proportional hazards models for each air pollutant or greenness, adjusting for all the aforementioned covariates. The equation is listed as follows:

(1) $$h\left(t|\left(Z_{i}\left(t\right),X_{i}\right)\right)=h_{0}\left(t\right)\times \exp\left(\gamma X_{i}+\beta Z_{i}\left(t\right)\right)$$

where *Z*_*i*_*(t)* stands for the time-varying exposures of air pollutants or NDVI for individual *i*. *X*_*i*_ denotes the baseline covariates mentioned above, except for age, which was treated as a time-varying covariate and calculated in one-year increments. The hazard function *h* at time *t* is influenced by the *Z*_*i*_*(t)* and *X*_*i*_ for each individual *i*, with *h*_*0*_ representing the baseline hazard function. The coefficients *γ* and *β* represent the effects of the covariate *X*_*i*_ and time-varying exposure *Z*_*i*_*(t)*, respectively. The models also accounted for within-individual correlation by specifying their unique participant IDs. These models were constructed with continuous exposures, accounting for per 10 μg/m^3^ increase for air pollutants, as well as per interquartile range (IQR) increase for NDVI. The results were reported as hazard ratios (HRs) with their respective 95% confidence intervals (CIs). We employed graphical diagnostics using Schoenfeld residuals to assess the proportional hazards assumption. The analysis indicated that the scaled residuals were not dependent on time (Supplementary Fig. S3), suggesting that the conditional proportional hazards assumption was satisfied.

To explore potential non-linearity in the impact of air pollution and greenness on both all-cause and cause-specific mortality, we utilised restricted cubic splines with four knots for air pollutants and three knots for NDVI.^22^^,^^41^^,^^42^ The final selection of knots was based on the Akaike Information Criterion (AIC) and Bayesian Information Criterion (BIC) values (Supplementary Table S2). Tests for non-linearity were conducted using Wald tests.

We hypothesised that mitigating air pollution could serve as a potential mechanism underlying the relationship between mortality and green space among individuals with diabetes. We therefore separated the overall impact of NDVI into non-mediated and mediated components,^43^ and conducted separate analyses to quantify the extent to which each air pollutant mediated greenness. Subsequently, we computed the proportion of the impact that each pollutant could account for, presenting the estimated mediation proportion with its 95% CI.^44^ Detailed methodology was provided in Supplementary Method S2.

To explore the effect modification between greenness and air pollutants, we first built the two-exposure models to regress both greenness and air pollution on mortality, and then added the product term of greenness and air pollution (i.e., NDVI × PM_2.5_ or NDVI × NO_2_) in the models to assess their interaction.

Several sensitivity analyses were performed. First, we developed two-pollutant models. Second, we performed additional adjustments for traffic noise, as it could potentially confound the observed relationships. This part of the analysis was restricted to the year 2019 when noise data was accessible. Third, we excluded cases that occurred within the first year of follow-up and reran the models to minimize potential early effects. Fourth, to explore the potential impact of the COVID-19 pandemic, we excluded the follow-up data from 2020 to 2021 and reran the models. Fifth, to assess the impact of different NDVI buffer sizes, we replaced the 250-m buffer NDVI with those at 500-m, 1-km, and 2-km buffers in the models. Sixth, we calculated E-values to assess the potential impact of unmeasured confounders on the observed associations. The E-value represents the minimum value of association that an unmeasured confounder would need to have with both the exposure and the outcome to fully explain the observed association.^45^ Seventh, we reran the models excluding smoking, alcohol use, and family history to assess the possible influences of their data missingness. Additionally, we employed the Fine and Gray subdistribution hazard model,^46^ to account for the competing risks of different causes of death. In this model, one specific cause was of primary interest, while other causes served as competing events. Lastly, to explore the potential differential effects of greenness across different areas, we stratified Shanghai into urban and suburban regions based on official divisions, and reran the models accordingly.

All statistical analyses were conducted using R software (version 4.3.0). A significance level of *P < 0.05* (two-tailed) was used to determine statistical significance.

### Role of funders

The funders had no role in the study design, data collection, data analysis, interpretation, or writing of the manuscript.

## Results

### Descriptive statistics

Table 1 presents the descriptive statistics of the characteristics and exposure levels of participants. A total of 174,063 participants with T2DM were included, with a median follow-up of 7.9 years (equivalent to 1,333,343 person-years). At baseline, the mean age was 64.4 years [standard deviation (SD): 10.9 years]. Approximately 52.9% of the participants were male, 55.9% had a normal BMI, 58.7% had no family history of diabetes, and 42.9% were employed. Notably, a significant portion of the study population demonstrated favorable health behaviors, with 70.2% reporting regular medication compliance, 62.1% engaging in regular physical activities, and 57.8% and 54.9% reporting never having smoked and consumed alcohol, respectively. The mean baseline total GDP (SD; ten thousand yuan) and the number of doctors per 10,000 people (SD) were 13,717 (4626) and 23.3 (15.2), respectively.

**Table 1:** The characteristics of participants and the exposure levels in the cohort of individuals with T2DM.

Variable	Overall	All causes	Non-death population
Number of patients	174,063	22,205	151,858
Total person year	1,333,343	142,776	1,190,567
Median of follow up	7.9	6.5	8.1
**Individual characteristic** (baseline)			
Age, mean (SD)	64.4 (10.9)	74.7 (10.3)	62.9 (10.2)
Sex (%)			
Female	47.1	45.3	46.1
Male	52.9	54.7	53.9
BMI (%)			
Normal	55.9	57.1	55.6
Underweight	3.9	5.9	3.7
Overweight	33.5	29.9	34.2
Obese	6.7	7.1	6.5
Family history (%)			
Yes	15.7	9.9	16.5
No	58.7	62.2	58.2
Unknown	25.6	27.9	25.3
Drink (%)			
Never	54.9	51.7	54.5
Sometimes^a^	9.8	9.1	9.8
Usual^b^	2.8	2.5	2.8
Every day	2.1	2.1	2.0
Unknown	30.4	34.6	30.9
Smoke (%)			
Never	57.8	54.4	57.4
Former smoker	2.4	3.0	2.2
Usual but not every day	1.4	1.2	1.4
Every day	8.0	6.8	8.1
Unknown	30.4	34.6	30.9
Sport (%)			
Yes	62.1	57.8	62.7
No	37.9	42.2	37.3
Medication (%)			
Regular	70.2	70.6	70.3
Irregular	2.4	2.7	2.4
None	27.4	26.7	27.3
Employment (%)			
Employed	42.9	43.0	42.9
Unemployed^c^	57.1	57.0	57.1
Total GDP, mean (SD), ten thousand *Yuan*	13,717 (4626)	13,815 (4415)	13,703 (4656)
The number of doctors per 10,000 people, mean (SD)	23.3 (15.2)	24.7 (15.8)	23.1 (15.1)
**Environmental exposures** [Median for the previous year of follow-up (P_25_, P_75_)]			
PM_2.5_ (μg/m^3^)	50.0 (40.2, 55.2)	51.2 (42.7, 56.2)	49.8 (40.0, 55.1)
PM_2.5-10_ (μg/m^3^)	32.9 (28.9, 42.7)	34.1 (29.7, 52.5)	32.7 (28.8, 42.3)
NO_2_ (μg/m^3^)	38.3 (34.8, 41.8)	38.7 (34.9, 42.4)	38.3 (34.7, 41.7)
NDVI (250-m buffer)	0.26 (0.22, 0.32)	0.25 (0.21, 0.30)	0.26 (0.22, 0.32)

BMI = body mass index; P_25_ = 25th percentile; P_75_ = 75th percentile; NDVI = normalized difference vegetation index.

a Sometimes = less than 1 day/month.

b Usual = 1 day/month to 6 days/week.

c Unemployed = participants who are unemployed, students, or retired.

The annual median concentrations (with 25th and 75th percentiles; μg/m^3^) of PM_2.5_, PM_2.5-10_, and NO_2_ for each participant were 50.0 (40.2, 55.2), 32.9 (28.9, 42.7), and 38.3 (34.8, 41.8), respectively. The annual median NDVI within a 250-m radius of residential areas was 0.26 (0.22, 0.32). Supplementary Figs. S4–S7 illustrate the spatial distributions of 5-year averages of air pollutants and NDVI across residential areas in Shanghai throughout the study period. The correlation coefficients among the pollutants ranged from −0.21 to 0.73, while those between each pollutant and NDVI ranged from −0.23 to −0.44 (Supplementary Table S3).

During the study period, we observed 22,205 cases of all-cause mortality, including CVD (8,997), IHD (4,105), stroke (4,332), respiratory disease (1,349), COPD (957), cancer (6,149), gastrointestinal cancer (3,058), lung cancer (1,468), metabolic disease (2,986), T2DM (2,912), and PVD (690).

### Regression results

**Fig. 1:**
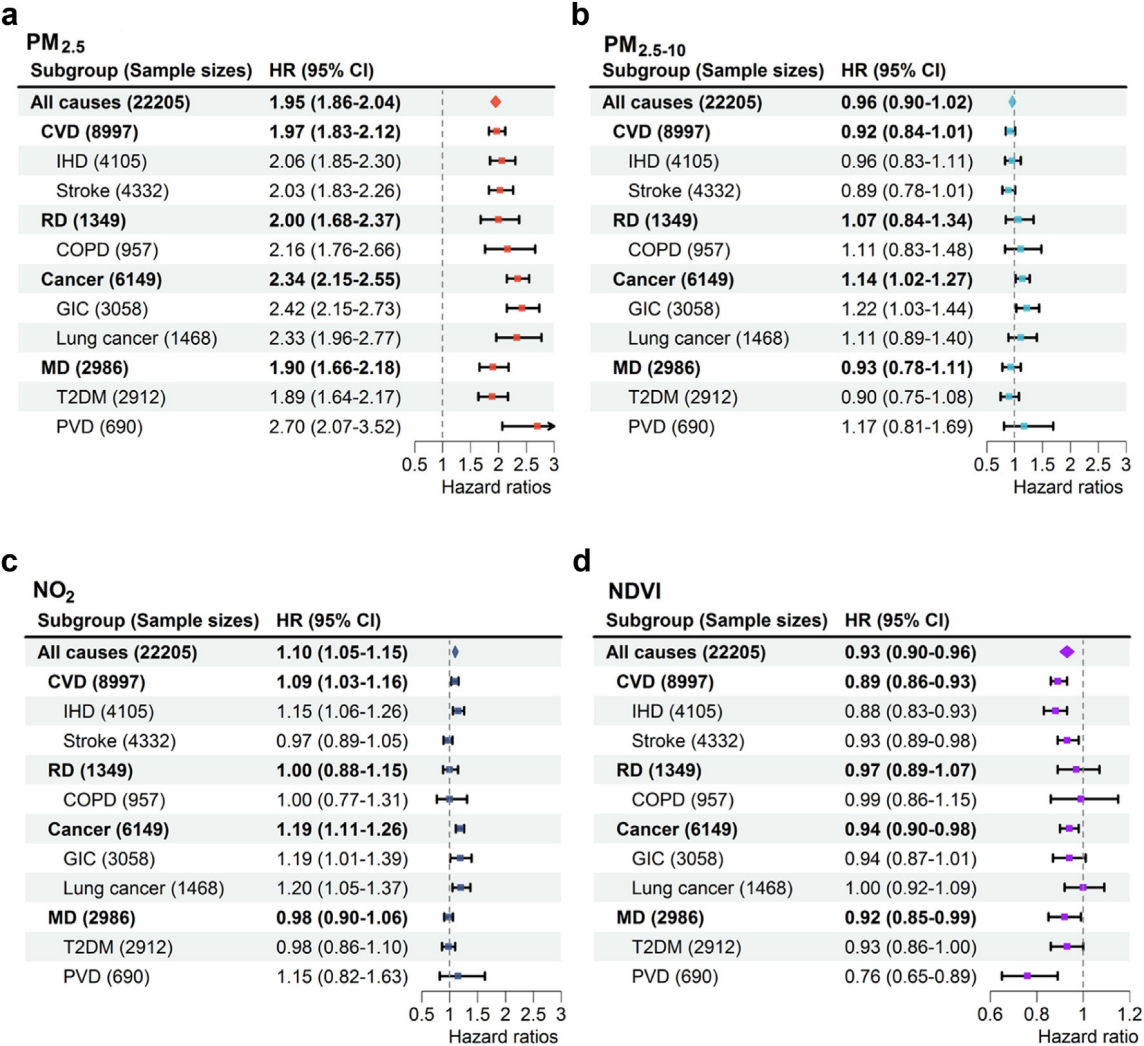
Adjusted HRs (95%CI) for all-cause and cause-specific mortality in participants with T2DM associated with long-term exposure to PM_2.5_, PM_2.5-10_, and NO_2_, as well as residential greenness (NDVI). Notes: COPD = chronic obstructive pulmonary disease; PVD = diabetes with peripheral vascular diseases; HR = hazard ratio; CI = confidence interval; NDVI = normalized difference vegetation index. HRs for mortality were related to each 10 μg/m^3^ increase in PM_2.5_, PM_2.5-10_, and NO_2_, and per interquartile range increase in NDVI (0.10), respectively.

Fig. 1 presents the adjusted HRs for all-cause and cause-specific mortality in T2DM associated with long-term exposure to air pollutants and NDVI. Specifically, long-term exposures to PM_2.5_ and NO_2_ were both significantly associated with increased all-cause mortality, with the effects of PM_2.5_ being more pronounced than those of NO_2_, while exposure to PM_2.5-10_ was not related to mortality. PM_2.5_ was consistently associated with heightened mortality from major causes (CVD, respiratory diseases, cancer, and metabolic disease), whereas PM_2.5-10_ only increased cancer mortality. Additionally, NO_2_ exposure was associated with increased mortality from CVD and cancer. For more specific causes, the most substantial effects of PM_2.5_ were observed in PVD mortality, followed by gastrointestinal cancer and lung cancer. Lung cancer and gastrointestinal cancer were the top causes of NO_2_-related mortality.

**Fig. 2:**
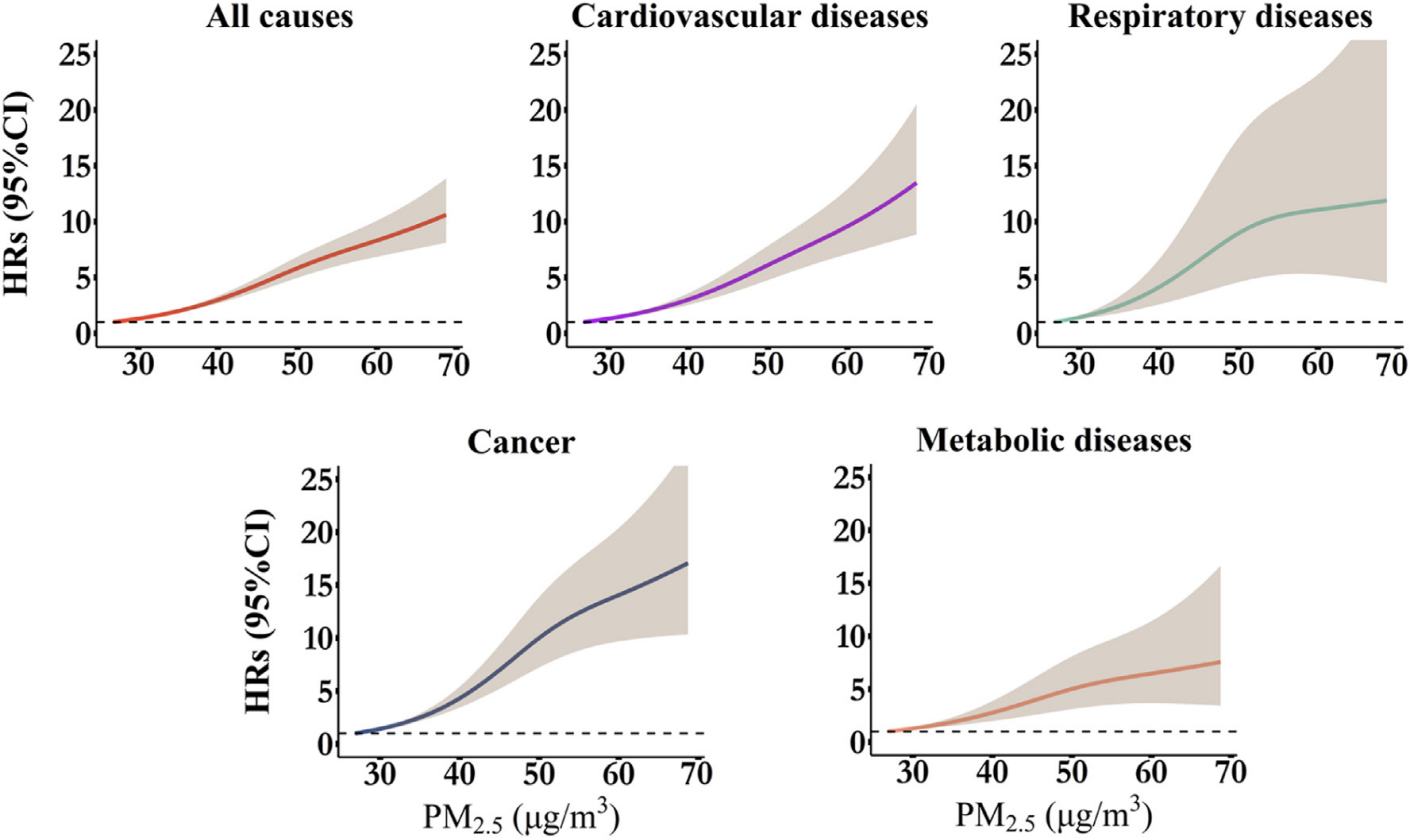
Nonlinear associations of PM_2.5_ with all-cause and cause-specific mortality in participants with T2DM. The models were adjusted for age, sex, body mass index, family history of diabetes, drinking status, smoking status, sports status, employed status, medication compliance, baseline comorbidities, total GDP, and district-level number of doctors. HR = hazard ratio; CI = confidence interval.

**Fig. 3:**
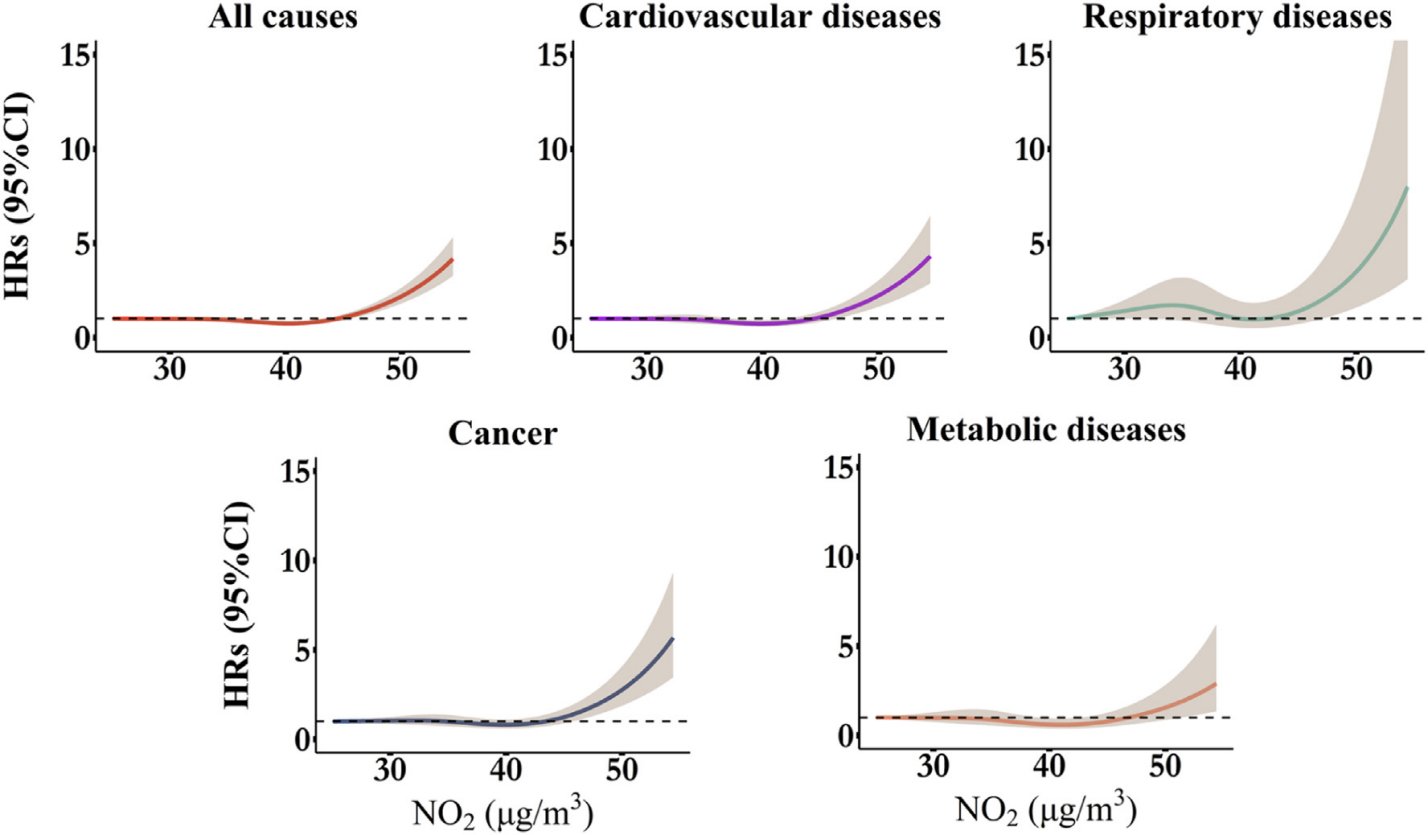
Nonlinear associations of NO_2_ with all-cause and cause-specific mortality in participants with T2DM. The models were adjusted for age, sex, body mass index, family history of diabetes, drinking status, smoking status, sports status, employed status, medication compliance, total GDP, and district-level number of doctors. HR = hazard ratio; CI = confidence interval.

The multivariable-adjusted restricted cubic splines for exposure-response relationships between air pollution and cause-specific mortality revealed a nearly linear pattern for PM_2.5_ and a non-linear pattern for NO_2_ (Fig. 2, Fig. 3). The risk associated with PM_2.5_ increased steadily with higher concentrations, while NO_2_ risks became significantly elevated only beyond concentrations of approximately 45 μg/m³. Due to the significant results in non-linearity tests (Supplementary Table S4), we also presented the risk estimates from models treating exposures as categorical variables, divided into quartiles. The stratified results were consistent with the overall exposure-response trends (Supplementary Table S5).

**Fig. 4:**
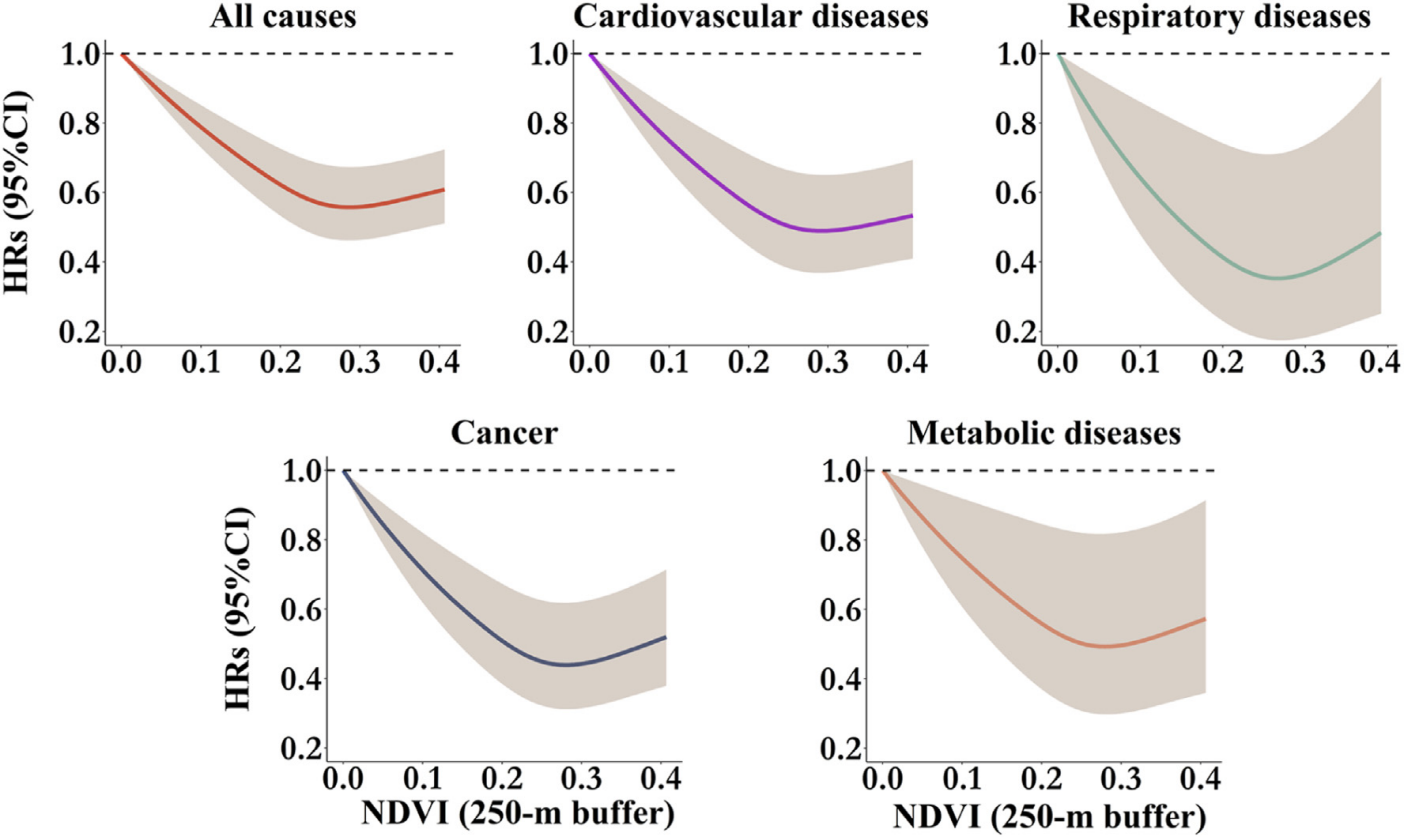
Nonlinear associations of residential greenness with all-cause and cause-specific mortality in participants with T2DM. The models were adjusted for age, sex, body mass index, family history of diabetes, drinking status, smoking status, sports status, employed status, medication compliance, total GDP, and district-level number of doctors. NDVI = normalized difference vegetation index; HR = hazard ratio; CI = confidence interval.

Regarding long-term exposure to greenness, an interquartile range (IQR) increase in NDVI (0.10) was linked to a significant reduction of 6%–24% in mortality risk across various causes in individuals with T2DM, except for respiratory diseases (Fig. 1). The most significant protective effects were observed for PVD mortality (HR: 0.76; 95% CI: 0.65, 0.89). The exposure-response curves for NDVI were consistent across different causes (Fig. 4), indicating an approximately linear pattern at low to moderate levels, but with a threshold after which the protective impacts slightly stabilized.

We observed significant mediation effects of air pollutants (PM_2.5_ and NO_2_) on the association between green spaces and all-cause mortality in populations with T2DM (Table 2). The reduction of PM_2.5_ and NO_2_ by green space might mediate 23.80% (8.91%, 32.43%) and 26.60% (2.39%, 50.81%) of the beneficial effect of residential green space, respectively. Beyond these mediation effects, we also found an interaction between NO_2_ and greenness on mortality risk (Table 2), with the HR for the interaction term being 0.84 (95% CI: 0.80, 0.89). However, no significant interaction effect was observed between PM_2.5_ and greenness [HR: 1.01 (95% CI: 0.98, 1.04)].

**Table 2:** The estimated proportion of associations between greenness and all-cause mortality mediated by air pollution (mediation analysis), and the association of the greenness and air pollution with all-cause mortality in participants with T2DM.

Analyses	Indicators	*P* values
**Mediation analysis**	**Mediation proportion (95% CI)**	
PM_2.5_	23.80% (8.91%, 32.43%)	0.002
NO_2_	26.60% (2.39%, 50.81%)	0.035

**Modification analysis**	**Hazard ratio (95% CI)**	

**Two-exposure model**		
NDVI	0.95 (0.92, 0.98)	<0.001
PM_2.5_	1.90 (1.81, 2.00)	<0.001
**Model with Interaction term**		
NDVI	0.90 (0.79, 1.01)	0.084
PM_2.5_	1.82 (1.65, 2.02)	<0.001
Interaction term	1.01 (0.98, 1.04)	0.348
**Two-exposure model**		
NDVI	0.95 (0.92, 0.98)	<0.001
NO_2_	1.10 (1.02, 1.18)	0.010
**Model with interaction term**		
NDVI	1.95 (1.60, 2.38)	<0.001
NO_2_	1.76 (1.51, 2.06)	<0.001
Interaction term	0.84 (0.80, 0.89)	<0.001

Notes: Hazard ratios for mortality were related to each 10 μg/m^3^ increase in PM_2.5_ and NO_2_, and per interquartile range increase in NDVI (0.10), respectively. The models were adjusted for age, sex, body mass index, family history of diabetes, drinking status, smoking status, sports status, employed status, medication compliance, total GDP, and district-level number of doctors.

NDVI = normalized difference vegetation index.

In sensitivity analyses, the associations between air pollution and mortality remained significant and stable in the two-pollutant model (Supplementary Table S6). When incorporating traffic noise, the relationships between air pollutants and green spaces and mortality were also stable (Supplementary Table S7). These associations did not significantly change after excluding participants whose outcomes occurred within the first year of follow-up (Supplementary Table S8). After excluding data from 2020 to 2021, the effect estimates slightly increased but the overall direction of the effects remained unchanged (Supplementary Table S9). The effects of NDVI exhibited a slight weakening trend as buffer sizes increase, but the direction remained consistent (Supplementary Table S10). According to E-values (Supplementary Table S11), the observed associations in this study are unlikely to be fully explained by unmeasured confounders. The inclusion of the “unknown” category did not significantly impact the overall associations (Supplementary Table S12). The competing risk analysis showed a decrease in the effect size, but the overall trends remained consistent (Supplementary Table S13). Lastly, the protective effects of greenness were significantly stronger for people with T2DM living in urban areas compared to those in suburban areas (Supplementary Table S14).

## Discussion

This 11-year prospective cohort study provides comprehensive insights into the associations of long-term exposure to air pollutants and green space with cause-specific mortality among individuals with T2DM. We observed significant associations of long-term exposure to PM_2.5_ with increased mortality across all causes, including CVDs, respiratory diseases, cancer, and metabolic diseases, while PM_2.5-10_ was only significantly related to cancer mortality. Long-term exposure to NO_2_ was also linked to heightened mortality, while the effects were less pronounced than those of PM_2.5_. The primary pollution-related mortality risks in people with T2DM stemmed from IHD, gastrointestinal cancer, lung cancer, and PVD. Our analysis revealed generally nonlinear or supra-linear exposure-response curves for air pollutants, with no discernible safety threshold for PM_2.5_. Additionally, long-term exposure to green spaces around residential areas was linked to decreased mortality risks across various diseases. The exposure-response analyses indicated a nearly linear protective effect on mortality at lower NDVI levels, with the protective impact partly attributed to reductions in PM_2.5_ and NO_2_ levels.

The estimated HRs for mortality linked to air pollutants in our study, particularly PM_2.5_, were notably higher than those reported in cohort studies of the general population,^8^, ^9^, ^10^, ^11^, ^12^ indicating a heightened sensitivity to air pollutants among patients with T2DM (Supplementary Table S15). Specifically, our HR estimates for PM_2.5_-related mortality substantially exceeded those reported in recent cohorts or meta-analyses.^8^, ^9^, ^10^, ^11^, ^12^ The HR for mortality associated with NO_2_ was also slightly higher than estimates from relevant cohort studies and meta-analyses.^9^, ^10^, ^11^, ^12^^,^^14^ Our results suggest that the adverse effects of particulate matter on individuals with T2DM are primarily driven by PM_2.5_ rather than PM_2.5-10_, highlighting the need to focus on PM_2.5_ control measures in regions with high diabetes prevalence. Furthermore, the observed non-linear exposure-response curves for NO_2_ in our study, which exhibit a clear risk threshold of about 45 μg/m³, suggest that targeted NO_2_ control measures up to a certain threshold could yield significant health benefits.

While previous cohort studies have explored the associations of air pollutants with cause-specific mortality in the general or elderly population,^6^, ^7^, ^8^, ^9^, ^10^, ^11^, ^12^, ^13^, ^14^ these connections in populations with diabetes remain largely unexplored. We observed more pronounced effects of air pollutants on diabetes complications (e.g., PVD) than on other mortality causes, possibly due to the role of oxidative stress in pollution-induced vascular dysfunction.^47^ Specifically, recent researches have indicated that air pollutants were related to chronic hyperglycemia, dyslipidemia, and blood pressure fluctuations in patients with diabetes,^48^^,^^49^ potentially triggering the release of inflammatory factors, generating reactive oxygen species, exacerbating abnormalities in endothelial function, and increasing the risk of both micro- and macrovascular diseases in this vulnerable group. Additionally, long-term exposure to both particulate matter and NO_2_ significantly increased the risk of cancer mortality in this population. This may be due to the disrupted glucose homeostasis by air pollutants,^48^^,^^49^ which in turn can elevate cancer risk.^50^

This study provides comprehensive evidence that residential greenness exposure may be a protective factor for individuals with T2DM. The HR for all-cause mortality related to green space in this population (0.93 per 0.1 increment of NDVI) is similar to HRs reported in various cohort studies of the general population, including those conducted in the USA (0.88),^18^ Spain (0.92),^16^ Canada (0.94),^24^ China (0.95),^22^ Switzerland (0.96),^21^ Australia (0.98),^23^ Italy (0.99),^26^ and estimates from meta-analyses (0.96).^20^ There are several potential biological explanations for this protective effect. Firstly, green spaces provide venues for physical activities, which is beneficial for individuals with T2DM in managing weight and improving insulin sensitivity.^51^ Secondly, green spaces promote relaxation and reduce stress, crucial for those with T2DM, as anxiety can elevate blood glucose levels and exacerbate symptoms.^52^ These explanations align with our stratified results based on urban and suburban NDVI data, as central urban residents typically experience higher work and life stress, lower physical activity levels, and higher pollutant exposure. The weaker protective effect of NDVI observed for residents in Shanghai’s suburban areas with higher levels of greenness may be attributed to their lower socioeconomic status, which offsets the benefits of greenness. This partially explains the flattening of the greenness–mortality curve at high NDVI levels. Thirdly, green spaces can enhance air quality by reducing concentrations of air pollutants,^43^ thereby decreasing pollution-related mortality. Our mediation analysis further supports this explanation. Additionally, the interaction between NO_2_ and greenness suggests that in areas with high green space coverage, the harmful effect of NO_2_ on mortality risk is mitigated. However, in areas with high NO_2_ pollution, the protective effect of green space is somewhat weakened. This underscores the need for a synergistic effort between strict emission control policies and the enhancement of green infrastructure. Moreover, the nearly linear exposure-response associations of green space with cause-specific mortality risks at low NDVI levels suggest that increasing green space in areas with low greenness may result in considerable health gains.

Our study had several strengths. First, we utilised a large-sample cohort of individuals with T2DM, which enables us to directly explore how air pollution affects this vulnerable population, rather than extrapolating the effects from the general population. Second, unlike studies conducted in European developed countries, the wide range of air pollutant distributions in our study offered a unique opportunity to investigate associations between air pollutants and cause-specific mortality in T2DM across the full exposure ranges. Third, the comprehensive exploration of the effects of green spaces provides not only a potential preventive measure to manage disease progression in individuals with diabetes but also evidence for public health policies on the synergistic effects of green spaces and pollutants.

Certain limitations warrant acknowledgement. First, despite employing high spatial resolutions for air pollution and NDVI, exposure misclassification remains possible due to the absence of personal continuous exposure monitoring and potential residential mobility during follow-up (albeit at a very low rate). Nevertheless, these errors are believed to be random and likely result in an underestimation of the effects.^53^ Second, NDVI provides a general measure of surrounding greenness without accounting for the quality or types of greenness, which might affect health outcomes through different mechanisms. Third, ozone concentrations inherently have much smaller spatial variations than PM_2.5_ and NO_2_ in our study region (Supplementary Table S16), limiting our ability to robustly assess the chronic health effects of ozone. Fourth, although this study has a large number of person-years of follow-up, the relatively short follow-up period may underestimate the long-term effects of air pollutants and NDVI. Lastly, residual confounding due to unmeasured factors (e.g., time-varying covariates and individual-level socioeconomic status) cannot be completely ruled out, despite the analysis using E-values indicating that unmeasured residual confounding is unlikely to entirely account for our observed associations.

This 11-year prospective cohort study provides compelling evidence linking particulate matter, particularly PM_2.5_ but not PM_2.5-10_, and NO_2_ to increased mortality risks of various causes in individuals with T2DM. These risks are generally beyond those observed in the general population and more likely to be attributed to diabetes complications, gastrointestinal cancer, lung cancer, and ischemic heart disease. Additionally, our study highlights the protective effect of green spaces on cause-specific mortality, partially mediated by improved air quality, especially in areas with low greenness. These findings support the need for policymakers, public health experts, and urban planners to develop greener, more sustainable neighbourhoods in regions with high air pollution and diabetes prevalence.

## Contributors

R.C., Y.S., and H.K. conceptualised and designed the study. C.W. and J.L. were responsible for the statistical analysis and drafted the manuscript. C.W. and J.L. also contributed to the visualisation of all figures and tables. X.M., J.C., Y.L., L.Q., R.G., J.F., and L.X. engaged in the interpretation of the data. R.C., Y.S., and H.K. reviewed and edited the manuscript. All authors had full access to and verified all the data underlying the study, and approved the final version of the manuscript.

## Data sharing statement

Patient-level data cannot be shared without approval from data custodians, due to local information governance and data protection regulations. Researchers can refer to the participants, who are listed as corresponding authors of this article, for information on the data upon reasonable request. Air pollution data can be accessed from the Methods section, which delineates specific descriptions of pollutants. NDVI data were obtained from MODIS Vegetation Index Products (https://modis.gsfc.nasa.gov/data/dataprod/mod13.php). GDP data were sourced from the National Tibetan Plateau/Third Pole Environment Data Center (https://data.tpdc.ac.cn/zh-hans/data/c91fd466-1f60-4bdb-8cdb-de6f663b0ae7/). The district-level availability rates of doctors were obtained from the Shanghai Statistical Yearbook (https://tjj.sh.gov.cn/tjnj/index_2.html).

## Declaration of interests

All authors declared no potential conflicts of interest.
