# Supplementary material for: Association of residential air pollution and green space with all-cause and cause-specific mortality in individuals with diabetes: an 11-year prospective cohort study
Source: eBioMedicine. 2024 Sep 30;108:105376. doi: 10.1016/j.ebiom.2024.105376 (PMC11472637; doi:10.1016/j.ebiom.2024.105376)
Supplement: Supplementary Methods S1 and S2, Figs. S1–S7, and Tables S1–S16 [file mmc1.docx]

**Supplementary materials**

**OUTLINE**

| **Title** |  | **Page** |
| --- | --- | --- |
| **Supplementary Methods** |  |  |
| **Supplementary Method S1:** The description of the Shanghai Standardized Diabetes Management System. |  | **1** |
| **Supplementary Method S2:** Mediation analysis. |  | **2** |
|  |  |  |
| **Supplementary Figures** |  |  |
| **Fig. S1.** Flow chart for study participant filtration. |  | **3** |
| **Fig. S2**. Directed Acyclic Graph for covariates selection and mediation analysis. |  | **4** |
| **Fig. S3.** Schoenfeld residual diagram for main models |  | **5** |
| **Fig. S4.** Spatial distribution of 5-year averages in concentration of PM_2.5_ across Shanghai, China. |  | **6** |
| **Fig. S5.** Spatial distribution of 5-year averages in concentration of PM_2.5-10_ across Shanghai, China. |  | **7** |
| **Fig. S6.** Spatial distribution of 5-year averages in concentration of NO_2_ across Shanghai, China. |  | **8** |
| **Fig. S7.** Spatial distribution of 5-year averages in NDVI (normalized difference vegetation index) across Shanghai, China. |  | **9** |
|  |  |  |
| **Supplementary Tables** |  |  |
| **Table S1.** Exposure levels in the cohort among included participants and those excluded due to missing covariates. |  | **10** |
| **Table S2.** AIC and BIC values for models of exposure and all-cause mortality fitted with restricted cubic splines using different numbers of knots. |  | **11** |
| **Table S3.** Correlations between environmental exposures. |  | **12** |
| **Table S4.** *P-values* for the non-linear tests. |  | **13** |
| **Table S5.** Adjusted HRs (95% CI) for all-cause and cause-specific mortality associated with long-term exposure to PM_2.5_, PM_2.5-10_, and NO_2_, as well as residential greenness (NDVI). All exposures were treated as categorial (four-factor quartile) variables. |  | **14** |

| **Table S6.** Results of 2-pollutant models. |  | **15** |
| --- | --- | --- |
| **Table S7.** Results of sensitivity analyses in the model including traffic noise. |  | **16** |
| **Table S8.** Results of sensitivity analyses in models using different sample selection. |  | **17** |
| **Table S9.** Adjusted HRs (95%CI) for all-cause and cause-specific mortality in patients with T2DM associated with long-term exposure to PM_2.5_, PM_2.5-10_, and NO_2_, as well as residential greenness (NDVI) **across 2011-2019**. |  | **18** |
| **Table S10.** Adjusted HRs (95%CI) for all-cause and cause-specific mortality in patients with T2DM associated with long-term exposure to residential greenness (NDVI) at different buffer sizes. |  | **19** |
| **Table S11.** E-values for various causes of mortality associated with PM_2.5_, NO_2_, and NDVI. |  | **20** |
| **Table S12.** Adjusted HRs (95%CI) for all-cause and cause-specific mortality in patients with T2DM associated with long-term exposure to air pollutants and residential greenness (NDVI) in models **without controlling** for smoking status, drinking frequency, and family history. |  | **21** |
| **Table S13**. Adjusted HRs (95%CI) for cause-specific mortality in patients with T2DM associated with long-term exposure to PM_2.5_, PM_2.5-10_, and NO_2_, as well as residential greenness (NDVI) in the Fine and Gray subdistribution hazard models. |  | **22** |
| **Table S14**. Adjusted hazard ratios (95%CI) for all-cause mortality in patients with T2DM associated with long-term exposure to residential greenness (NDVI) in urban and suburban regions of Shanghai. |  | **23** |
| **Table S15.** Comparison of findings for natural mortality with ESCAPE, ESCAPE, recent North American administrative cohorts, and recent meta-analyses estimates in general population. |  | **24** |
| **Table S16.** Distribution of air pollution exposure at participant addresses in our cohort across 2010-2020. |  | **25** |

**Supplementary Methods**

**Supplementary Method S1: The description of the Shanghai Standardized Diabetes Management System.**

The Shanghai Municipal Center for Disease Control and Prevention (SCDC) initiated the community diabetes management information system in 2004, gradually expanding to 33 community health service centers. After the launch of the National Basic Public Health Service Program (NBPHSP) in 2009, the system expanded to cover 241 community health service centers across 16 districts of Shanghai.

Patients with type 2 diabetes (diagnosed by FBG and 2hBG according to the WHO 1998 diagnostic criteria) are registered into the Shanghai standardized diabetes management system (DSSDMS) by community general practitioners. This registration is part of the national basic public health service, which also includes follow-up management.

Community health centers (CHCs) in Shanghai are responsible for managing patients with T2DM and uploading electronic records to the DSSDMS. All diagnosed cases of T2DM, including those identified through community-based screenings, physical examinations, and routine outpatient visits, are required to be registered in the system. Participants are identified as having T2DM if their FBG level is ≥ 7.0 mmol/L and/or their 2-hour glucose level (after a 75 g oral glucose tolerance test) is ≥ 11.1 mmol/L, or if they are using antidiabetic medication according to WHO criteria.

To ensure the accuracy and reliability of the registration management information, the SCDC implemented a quality control process. This involved the random selection of a proportion of patients with T2DM across the 16 district CDCs, followed by annual telephone and face-to-face investigations.

**Supplementary Method S2: Mediation analysis**.

We hypothesized that mitigating air pollution could serve as a potential mechanism underlying the relationship between mortality and green space among individuals with diabetes. Specifically, we separated the overall impact of NDVI into non-mediated and mediated components. When PM_2.5_ is considered a mediator, the overall impact indicates the average mortality shift across the population if NDVI values changed from the baseline to another value. The non-mediated component reflects the variation in mortality if NDVI values shifted from 0 to 1 while maintaining PM_2.5_ concentration constant at its natural level for an individual (i.e., excluding PM_2.5_ effects). Conversely, the mediated component signifies the average mortality change if PM_2.5_ levels shifted from their natural state to another level while NDVI was fixed at a value like 1.

We conducted separate analyses to quantify the extent to which each air pollutant mediated greenness based on the main models. For each pollutant, we developed two distinct models: one for the pollutant and another for the mortality. In the pollutant model (a linear regression model), the pollutant was regressed on the NDVI and covariates. In the mortality model (a time-dependent Cox model), the mortality was regressed on the NDVI, pollutant, and covariates. We derived the mediated and non-mediated impacts from the pollutant and mortality model estimates, respectively. Subsequently, we computed the proportion of the impact that each pollutant could account for and presented the estimated mediation proportion along with its 95% CI.

**Supplementary Fig.s**


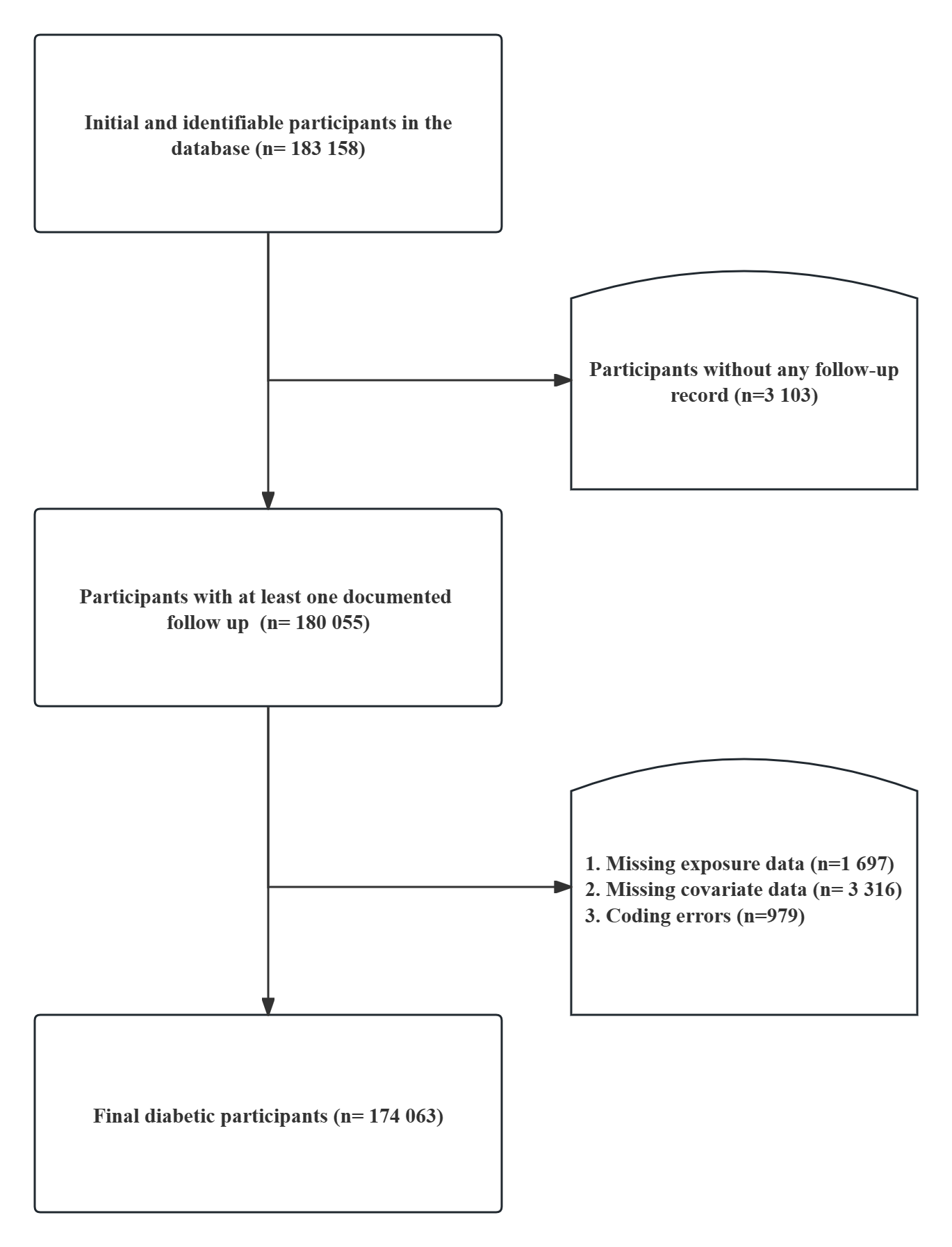


**Fig. S1.** Flow chart for study participant filtration.


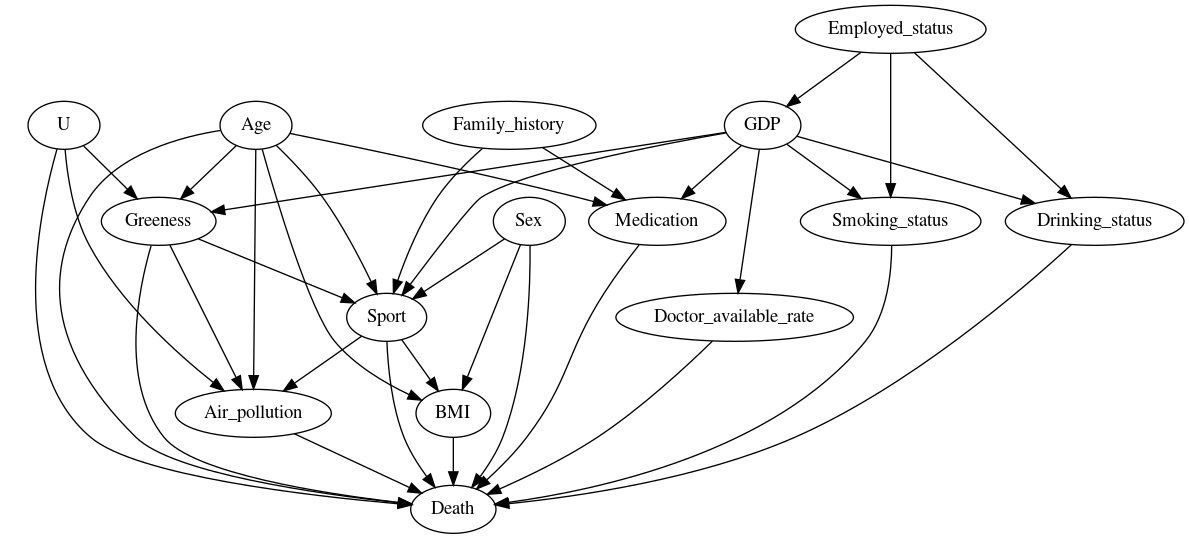


**Fig. S2.** Directed Acyclic Graph for covariates selection and mediation analysis. Notes: U refers to unobserved variables.

**

**

**Fig. S3.** Schoenfeld residual diagram for main models.


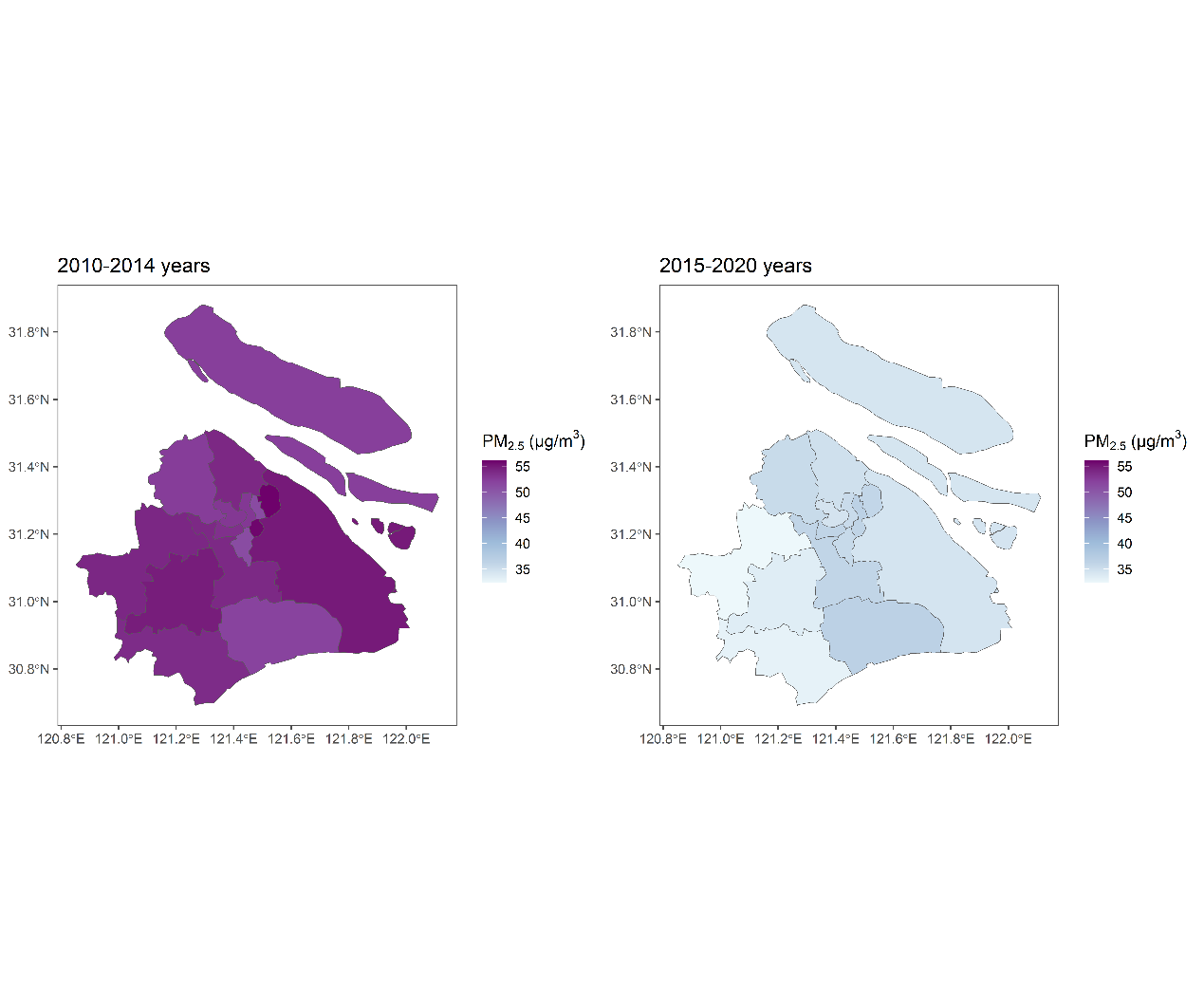


**Fig. S4.** Spatial distribution of 5-year averages in concentration of PM_2.5_ across Shanghai, China.


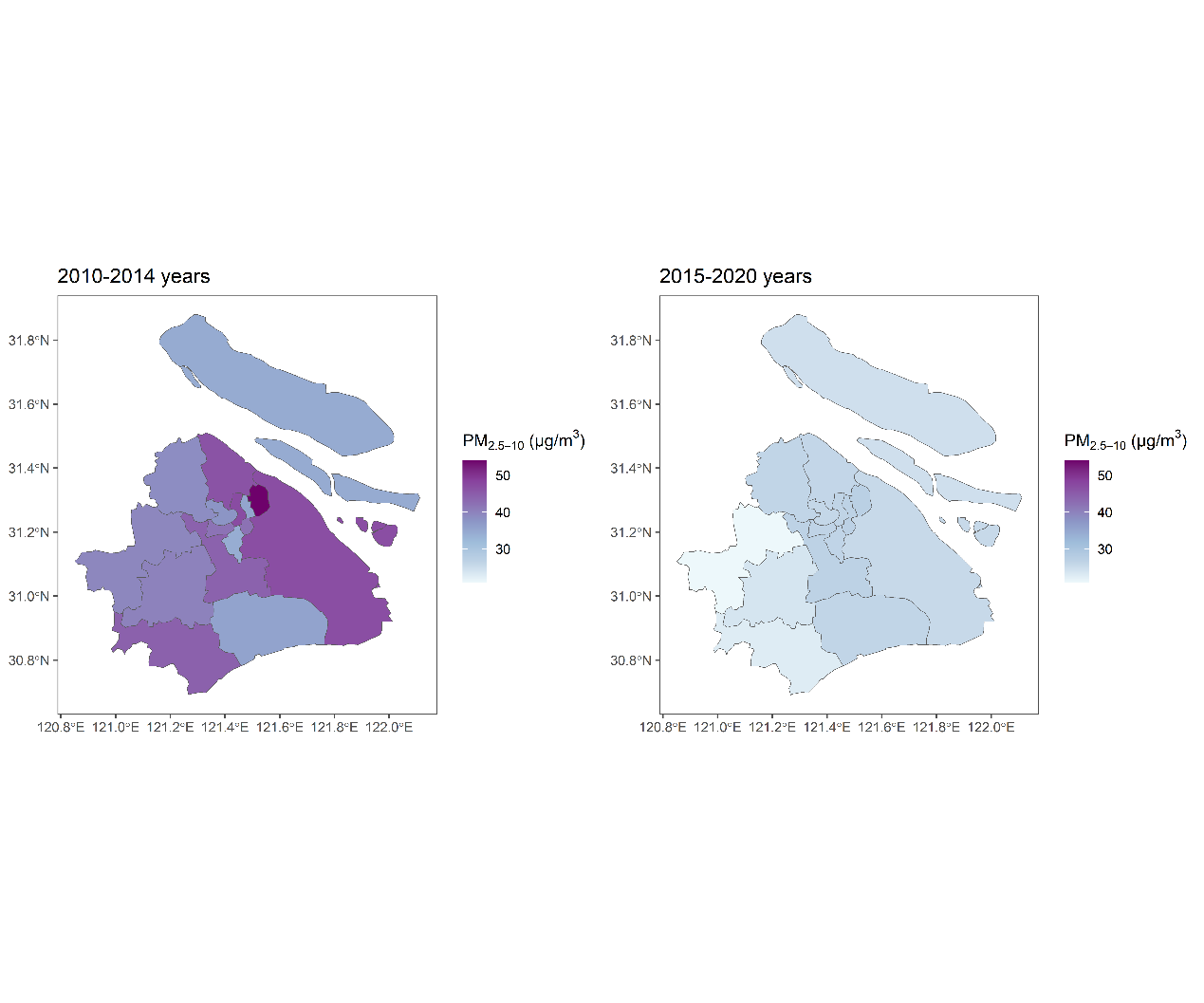


**Fig. S5.** Spatial distribution of 5-year averages in concentration of PM_2.5-10_ across Shanghai, China.


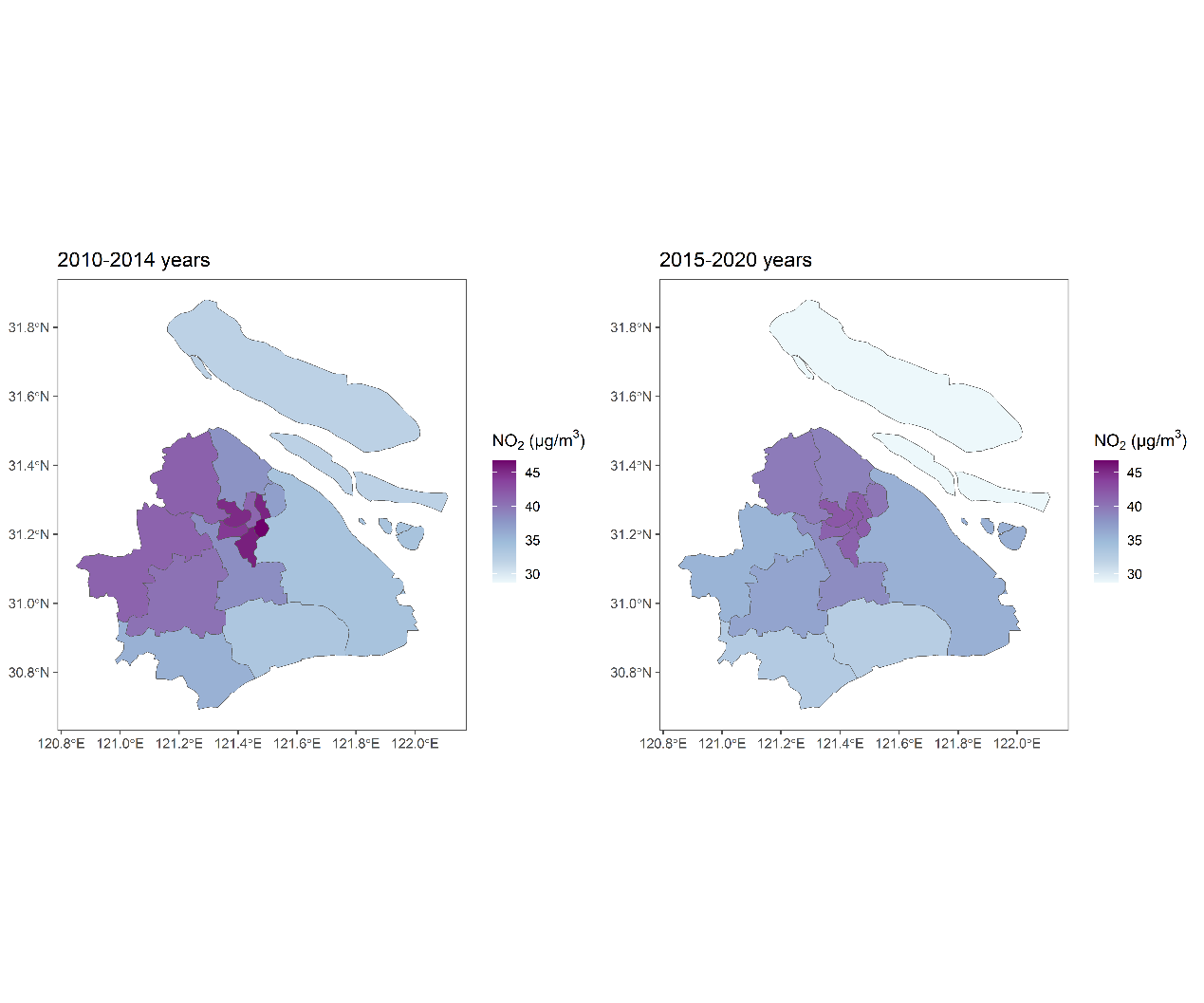


**Fig. S6.** Spatial distribution of 5-year averages in concentration of NO_2_ across Shanghai, China.


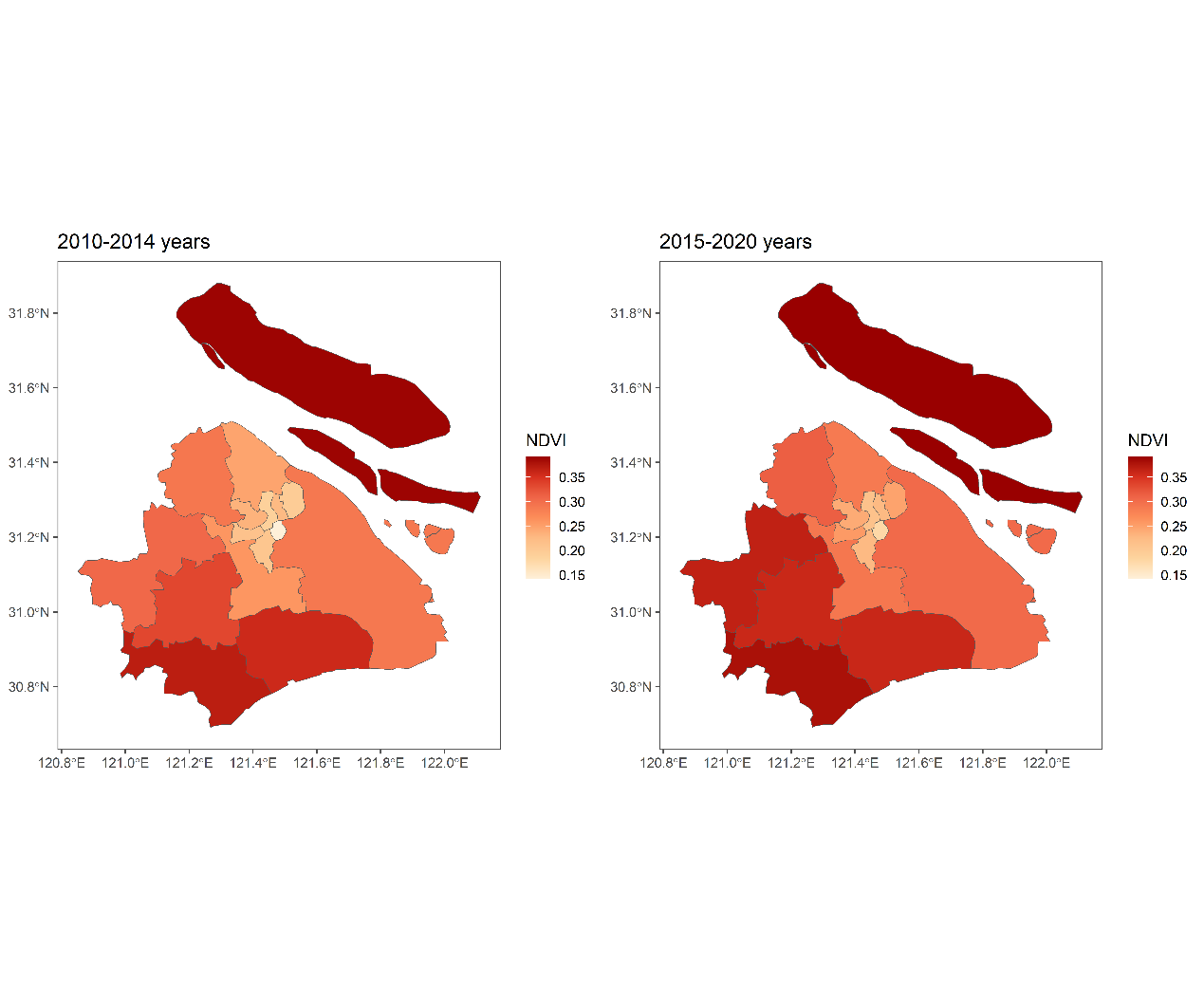


**Fig. S7.** Spatial distribution of 5-year averages in NDVI (normalized difference vegetation index) across Shanghai, China.

**Supplementary Tables**

**Table S1.** Exposure levels in the cohort among included participants and those excluded due to missing covariates.

| **Exposure** | **Included participants (N=174,063)** | **Excluded participants (N=3,316)** |
| --- | --- | --- |
| **PM_2.5_** | 50.0 (40.2, 55.2) | 49.9 (40.3, 55.1) |
| **PM_2.5-10_** | 32.9 (28.9, 42.7) | 33.0 (29.0, 43.1) |
| **NO_2_** | 38.3 (34.8, 41.8) | 38.1 (34.5, 41.2) |
| **NDVI** | 0.26 (0.22, 0.32) | 0.26 (0.22, 0.31) |

Notes: Values in the cells refer to median (P25, P75) for the previous year of follow-up.

**Table S2.** AIC and BIC values for models of exposure and all-cause mortality fitted with restricted cubic splines using different numbers of knots.

| Exposures | Knots=3 | | Knots=4 | |
| --- | --- | --- | --- | --- |
|  | AIC | BIC | AIC | BIC |
| PM_2.5_ | 313,592 | 313,900 | **312,790** | **313,109** |
| NO_2_ | 317,317 | 317,624 | **317,189** | **317,508** |
| NDVI | **288,894** | **289,152** | 288,895 | 289,165 |

Notes: AIC=Akaike Information Criterion; BIC=Bayesian Information Criterion.

**Table S3.** Correlations between environmental exposures.

| Exposure | PM_2.5_ | PM_2.5-10_ | NO_2_ | NDVI |
| --- | --- | --- | --- | --- |
| PM_2.5_ | 1.00 |  |  |  |
| PM_2.5-10_ | 0.73 | 1.00 |  |  |
| NO_2_ | 0.13 | -0.21 |  |  |
| NDVI | -0.23 | -0.28 | -0.44 | 1.00 |

Notes: The values in cells are Spearman’s correlation coefficients. NDVI=normalized difference vegetation index.

**Table S4.** *P-values* for the non-linear tests.

| **Causes** | **PM_2.5_** | **NO_2_** | **NDVI** |
| --- | --- | --- | --- |
| **All cause** | <0.001 | <0.001 | <0.001 |
| **CVD** | 0.002 | <0.001 | <0.001 |
| **Respiratory disease** | 0.007 | <0.001 | 0.002 |
| **Cancer** | <0.001 | <0.001 | <0.001 |
| **Metabolic disease** | 0.042 | <0.001 | <0.001 |

Notes: CVD=cardiovascular disease; RD=respiratory disease; MD=metabolic disease.

**Table S5.** Adjusted HRs (95% CI) for all-cause and cause-specific mortality associated with long-term exposure to PM_2.5_, PM_2.5-10_, and NO_2_, as well as residential greenness (NDVI). All exposures were treated as categorial (four-factor quartile) variables.

|  | **All causes** | **CVD** | **RD** | **Cancer** | **MD** |
| --- | --- | --- | --- | --- | --- |
| **PM_2.5_** |  |  |  |  |  |
| Q1 | Ref | Ref | Ref | Ref | Ref |
| Q2 | 2.46 (2.35, 2.57) | 2.48 (2.33, 2.63) | 2.82 (2.42, 3.29) | 2.89 (2.69, 3.12) | 2.43 (2.13, 2.78) |
| Q3 | 2.95 (2.65, 3.26) | 2.85 (2.41, 3.30) | 3.30 (2.33, 4.42) | 4.42 (3.78, 5.13) | 2.27 (1.36, 3.30) |
| Q4 | 4.34 (4.00, 4.70) | 4.25 (3.76, 4.78) | 5.11 (3.92, 6.41) | 5.75 (4.78, 6.82) | 4.23 (3.45, 5.43) |
| **PM_2.5_10_** |  |  |  |  |  |
| Q1 | Ref | Ref | Ref | Ref | Ref |
| Q2 | 0.99 (0.93, 1.05) | 0.92 (0.85, 1.00) | 1.10 (0.91, 1.33) | 1.11 (1.02, 1.21) | 1.02 (0.88, 1.18) |
| Q3 | 1.00 (0.91, 1.10) | 1.12 (0.99, 1.25) | 1.07 (0.74, 1.46) | 1.37 (1.21, 1.54) | 1.02 (0.79, 1.29) |
| Q4 | 0.89 (0.65, 1.17) | 0.94 (0.68, 1.54) | 1.96 (0.87, 3.26) | 1.52 (1.06, 2.17) | 0.58 (0.22, 1.05) |
| **NO_2_** |  |  |  |  |  |
| Q1 | Ref | Ref | Ref | Ref | Ref |
| Q2 | 1.00 (0.95, 1.05) | 1.04 (0.99, 1.09) | 1.00 (0.90, 1.12) | 1.02 (0.96, 1.08) | 0.94 (0.81, 1.09) |
| Q3 | 0.95 (0.91, 0.99) | 0.95 (0.91, 0.99) | 0.99 (0.92, 1.06) | 1.00 (0.94, 1.04) | 0.86 (0.76, 0.98) |
| Q4 | 1.07 (1.01, 1.14) | 1.08 (1.02, 1.15) | 1.10 (0.92, 1.29) | 1.18 (1.10, 1.26) | 0.99 (0.85, 1.14) |
| **NDVI** |  |  |  |  |  |
| Q1 | Ref | Ref | Ref | Ref | Ref |
| Q2 | 0.92 (0.88, 0.96) | 0.90 (0.85, 0.95) | 0.83 (0.73, 0.95) | 0.86 (0.81, 0.91) | 0.95 (0.84, 1.07) |
| Q3 | 0.90 (0.85, 0.94) | 0.88 (0.83, 0.92) | 0.78 (0.68, 0.90) | 0.85 (0.80, 0.91) | 0.87 (0.77, 0.98) |
| Q4 | 0.90 (0.86, 0.96) | 0.85 (0.80, 0.89) | 0.95 (0.83, 1.08) | 0.90 (0.85, 0.95) | 0.95 (0.82, 1.10) |

Notes: CVD=cardiovascular disease; RD=respiratory disease; MD=metabolic disease.

Q1–Q4 refer to the following quartiles: below the 25th percentile, the 25th to 50th percentile, the 50th to 75th percentile, and above the 75th percentile, respectively.

**Table S6.** Results of 2-pollutant models.

| Exposure | Model | HRs for exposure (95%CI) | HRs for NO_2_ (95%CI) |
| --- | --- | --- | --- |
| PM_2.5_ | + NO_2_ | 1.98 (1.89, 2.07) | 1.07 (1.01, 1.14) |
| PM_2.5-10_ | + NO_2_ | 0.95 (0.90, 1.01) | 1.12 (1.06, 1.18) |

Notes: HRs (95% CI) are results for per 10 μg/m^3^ increase in PM_2.5_, PM_2.5-10_, and NO_2_. HR=hazard ratio; CI=confidence interval.

**Table S7.** Results of sensitivity analyses in the model including traffic noise.

| Exposure | HRs (95%CI) |
| --- | --- |
| PM_2.5_ | 2.55 (2.43, 2.67) |
| PM_2.5-10_ | 0.92 (0.82, 1.01) |
| NO_2_ | 1.09 (1.02, 1.16) |
| NVDI | 0.95 (0.91, 0.99) |

Notes: HRs for mortality were related to each 10 μg/m^3^ increase in PM_2.5_, PM_2.5-10_, and NO_2_, and per interquartile range increase in NDVI (0.10), respectively. HR=hazard ratio; CI=confidence interval. This part of analysis was restricted to the year of 2019, when noise data was accessible. **Table S8.** Results of sensitivity analyses in models using different sample selection.

| Exposure | HRs (95%CI) |
| --- | --- |
| PM_2.5_ | 1.95 (1.86, 2.05) |
| PM_2.5-10_ | 0.97 (0.91, 1.03) |
| NO_2_ | 1.09 (1.03, 1.15) |
| NVDI | 0.94 (0.92, 0.97) |

**Notes:** Exclusion of mortality cases within the initial years of follow-up.

The values in cells are hazard ratios with their 95% confidence interval.

**Table S9.** Adjusted HRs (95%CI) for all-cause and cause-specific mortality in patients with T2DM associated with long-term exposure to PM_2.5_, PM_2.5-10_, and NO_2_, as well as residential greenness (NDVI) **across 2011-2019**.

| **Causes** | **PM_2.5_** | **PM_2.5-10_** | **NO_2_** | **NDVI** |
| --- | --- | --- | --- | --- |
| **All cause** | 2.06 (1.93, 2.19) | 0.97 (0.89, 1.06) | 1.16 (1.07, 1.26) | 0.91 (0.87, 0.94) |
| **CVD** | 2.16 (1.95, 2.38) | 0.97 (0.83, 1.12) | 1.16 (1.01, 1.32) | 0.88 (0.83, 0.93) |
| **RD** | 2.15 (1.81, 2.54) | 1.17 (0.90, 1.48) | 1.10 (0.85, 1.34) | 0.93 (0.86, 1.02) |
| **Cancer** | 2.64 (2.43, 2.87) | 1.26 (1.09, 1.42) | 1.32 (1.12, 1.55) | 0.90 (0.85, 0.96) |
| **MD** | 2.27 (1.99, 2.59) | 1.13 (0.88, 1.39) | 1.00 (0.78, 1.27) | 0.91 (0.83, 0.99) |

Note:The values in cells are hazard ratios with their 95% confidence interval. CVD=cardiovascular disease; RD=respiratory disease; MD=metabolic disease.

**Table S10.** Adjusted HRs (95%CI) for all-cause and cause-specific mortality in patients with T2DM associated with long-term exposure to residential greenness (NDVI) at different buffer sizes.

| **Causes** | **NDVI (500-m)** | **NDVI (1000-m)** | **NDVI (2000-m)** |
| --- | --- | --- | --- |
| **All cause** | 0.94 (0.92, 0.97) | 0.94 (0.91, 0.97) | 0.95 (0.92, 0.98) |
| **Cardiovascular disease** | 0.91 (0.87, 0.95) | 0.91 (0.87, 0.95) | 0.93 (0.88, 0.97) |
| Ischemic heart disease | 0.88 (0.82, 0.94) | 0.88 (0.83, 0.94) | 0.92 (0.85, 0.99) |
| Stroke | 0.94 (0.89, 0.99) | 0.94 (0.88, 1.00) | 0.96 (0.89, 1.03) |
| **Respiratory disease** | 0.97 (0.86, 1.09) | 0.96 (0.86, 1.07) | 0.94 (0.82, 1.06) |
| COPD | 0.99 (0.87, 1.14) | 0.98 (0.86, 1.12) | 0.98 (0.84, 1.15) |
| **Cancer** | 0.94 (0.90, 0.99) | 0.94 (0.90, 0.99) | 0.95 (0.89, 1.00) |
| Gastrointestinal cancer | 0.94 (0.87, 1.01) | 0.94 (0.87, 1.00) | 0.95 (0.88, 1.04) |
| Lung cancer | 1.01 (0.91, 1.13) | 1.02 (0.92, 1.14) | 1.05 (0.93, 1.20) |
| **Metabolic disease** | 0.92 (0.85, 0.99) | 0.94 (0.88, 1.02) | 0.94 (0.86, 1.02) |
| Type 2 Diabetes | 0.92 (0.85, 1.00) | 0.94 (0.88, 1.02) | 0.95 (0.87, 1.03) |
| PVD | 0.77 (0.66, 0.90) | 0.76 (0.65, 0.89) | 0.76 (0.65, 0.88) |

Notes: HRs for mortality were related to per interquartile range increase in NDVI (0.10), respectively. HR=hazard ratio; CI=confidence interval.

**Table S11.** E-values for various causes of mortality associated with PM_2.5_, NO_2_, and NDVI.

| **Causes** | **PM_2.5_** | | **NO_2_** | | **NDVI** | |
| --- | --- | --- | --- | --- | --- | --- |
|  | **Point^*^** | **Lower^**^** | **Point** | **Lower** | **Point** | **Lower** |
| **All cause** | 3.31 | 3.12 | 1.43 | 1.28 | 1.36 | 1.25 |
| **Cardiovascular disease** | 3.35 | 3.06 | 1.40 | 1.21 | 1.50 | 1.36 |
| IHD | 3.54 | 3.10 | 1.57 | 1.31 | 1.53 | 1.36 |
| Stroke | 3.48 | 3.06 | No applicable | | 1.36 | 1.16 |
| **Respiratory disease** | 3.41 | 2.75 | No applicable | | No applicable | |
| COPD | 3.74 | 2.92 | No applicable | | No applicable | |
| **Cancer** | 4.11 | 3.72 | 1.67 | 1.46 | 1.32 | 1.16 |
| GI cancer | 4.27 | 3.72 | No applicable | | No applicable | |
| Lung cancer | 4.09 | 3.33 | 1.67 | 1.28 | No applicable | |
| **Metabolic disease** | 3.21 | 2.71 | No applicable | | 1.39 | 1.11 |
| Type 2 Diabetes | 3.19 | 2.66 | No applicable | | No applicable | |
| PVD | 4.84 | 3.56 | No applicable | | 1.96 | 1.50 |

*Point Estimate E-value.

** Lower Bound E-value.

**Table S12.** Adjusted HRs (95%CI) for all-cause and cause-specific mortality in patients with T2DM associated with long-term exposure to air pollutants and residential greenness (NDVI) in models **without controlling** for smoking status, drinking frequency, and family history.

| **Causes** | **PM_2.5_** | **PM_2.5-10_** | **NO_2_** | **NDVI** |
| --- | --- | --- | --- | --- |
| **All cause** | 1.94 (1.85, 2.03) | 0.97 (0.89, 1.06) | 1.09 (1.04, 1.16) | 0.95 (0.92, 0.98) |
| **CVD** | 1.96 (1.82, 2.11) | 0.93 (0.84, 1.03) | 1.10 (1.01, 1.19) | 0.91 (0.87, 0.95) |
| **RD** | 1.99 (1.67, 2.36) | 1.09 (0.86, 1.37) | 1.00 (0.80, 1.25) | 0.98 (0.87, 1.10) |
| **Cancer** | 2.32 (2.13, 2.54) | 1.18 (1.06, 1.32) | 1.18 (1.06, 1.31) | 0.95 (0.90, 1.00) |
| **MD** | 1.90 (1.66, 2.17) | 0.91 (0.77, 1.09) | 0.99 (0.87, 1.13) | 0.96 (0.88, 1.03) |

Notes: The values in cells are hazard ratios with their 95% confidence interval. CVD=cardiovascular disease; RD=respiratory disease; MD=metabolic disease.

**Table S13**. Adjusted hazard ratios (95%CI) for cause-specific mortality in patients with T2DM associated with long-term exposure to PM_2.5_, PM_2.5-10_, and NO_2_, as well as residential greenness (NDVI) in the Fine and Gray subdistribution hazard models.

| **Causes** | **PM_2.5_** | **NO_2_** | **NDVI** |
| --- | --- | --- | --- |
| **Cardiovascular disease** | 1.88 (1.74, 2.04) | 1.03 (0.93, 1.15) | 0.94 (0.90, 0.98) |
| **Respiratory disease** | 1.26 (1.14, 1.38) | 0.89 (0.69, 1.14) | 1.00 (0.86, 1.15) |
| **Cancer** | 2.00 (1.83, 2.19) | 1.08 (1.00, 1.16) | 0.95 (0.90, 1.00) |
| **Metabolic disease** | 1.58 (1.37, 1.81) | 0.96 (0.42, 1.72) | 0.99 (0.90, 1.09) |

Notes: The values in cells are hazard ratios with their 95% confidence interval.

**Table S14**. Adjusted hazard ratios (95%CI) for all-cause mortality in patients with T2DM associated with long-term exposure to residential greenness (NDVI) in urban and suburban regions of Shanghai.

| **Regions** | **Median (P_25_, P_75_)^#^** | **Hazard ratios (95% CI)** |
| --- | --- | --- |
| **Urban** | 0.21 (0.18, 0.25) | 0.90 (0.86, 0.94) |
| **Suburban** | 0.30 (0.25, 0.37) | 0.98 (0.94, 1.03) |

**^#^** Median for the previous year of follow-up; P_25_=25th percentile; P_75_=75th percentile;

Notes: 95% CI=95% confidence interval.

**Table S15.** Comparison of findings for natural mortality with ESCAPE, ESCAPE, recent North American administrative cohorts, and recent meta-analyses estimates in general population.

| **Cohort/Meta analysis** | **PM_2.5_** | **NO_2_** |
| --- | --- | --- |
| ESCAPE^1^ | 1.14 (1.04, 1.26) | 1.01 (0.99, 1.03) |
| ELAPSE^2^ | 1.28 (1.22, 1.33) | 1.09 (1.07, 1.10) |
| CanCHEC^3^ | 1.07 (1.06, 1.08) | 1.06 (1.05, 1.07) |
| MEDICARE cohort^4^ | 1.07 (1.07, 1.08) |  |
| Danish cohort^5^ | 1.08 (1.04, 1.13) | 1.05 (1.04, 1.06) |
| Meta analysis^6^ | 1.08 (1.06, 1.09) |  |
| Meta analysis^7^ |  | 1.02 (1.01, 1.04) |

Notes: The values in cells are hazard ratios with their 95% confidence interval for mortality in relation to per 10 μg/m^3^ increase in PM_2.5_ and NO_2_, respectively.

Reference

1. Beelen R, Raaschou-Nielsen O, Stafoggia M, et al. Effects of long-term exposure to air pollution on natural-cause mortality: an analysis of 22 European cohorts within the multicentre ESCAPE project. Lancet 383:785-795, 2014.

2. Strak M, Weinmayr G, Rodopoulou S, et al. Long term exposure to low level air pollution and mortality in eight European cohorts within the ELAPSE project: pooled analysis. BMJ 374:n1904, 2021.

3. Crouse DL, Peters PA, Hystad P, ET AL. Ambient PM2.5, O(3), and NO(2) Exposures and Associations with Mortality over 16 Years of Follow-Up in the Canadian Census Health and Environment Cohort (CanCHEC). Environ Health Perspect 123:1180-1186, 2015.

4. Di Q, Wang Y, Zanobetti A, Wang Y, et al. Air Pollution and Mortality in the Medicare Population. N Engl J Med 376:2513-2522, 2017.

5. Raaschou-Nielsen O, Thorsteinson E, Antonsen S, et al. Long-term exposure to air pollution and mortality in the Danish population a nationwide study. EClinicalMedicine 28:100605, 2020.

6. Chen J, Hoek G. Long-term exposure to PM and all-cause and cause-specific mortality: A systematic review and meta-analysis. Environ Int 143:105974, 2020.

7. Huangfu P, Atkinson R. Long-term exposure to NO(2) and O(3) and all-cause and respiratory mortality: A systematic review and meta-analysis. Environ Int 144:105998, 2020.

**Table S16.** Distribution of air pollution exposure at participant addresses in our cohort across 2010-2020.

| **Indicators** (μg/m^3^) | **PM_2.5_** | **PM_2.5-10_** | **NO_2_** | **O_3_** |
| --- | --- | --- | --- | --- |
| Mean | 47.8 | 37.2 | 38.4 | 100.9 |
| **SD** | **9.4** | **11.4** | **5.2** | **5.3** |
| Min | 25.0 | 15.5 | 16.5 | 68.6 |
| **P5** | **32.9** | **24.7** | **30.2** | **94.5** |
| P25 | 40.2 | 28.9 | 34.8 | 96.9 |
| **P50** | **50.0** | **32.9** | **38.3** | **99.4** |
| P75 | 55.2 | 42.7 | 41.8 | 104.2 |
| **P95** | **62.1** | **57.6** | **47.2** | **110.9** |
| Max | 85.6 | 69.6 | 57.5 | 118.3 |

Notes: SD=standard deviation.
